# Malignant Eccrine Adenoma With Sarcomatous (Heterologous) Components: Report of a Rare Skin Adnexal Neoplasm With Literature Review

**DOI:** 10.7759/cureus.12390

**Published:** 2020-12-30

**Authors:** Hira Ishtiaq, Muhammad Abdulwaasey, Muhammad Usman Tariq, Saira Fatima

**Affiliations:** 1 Histopathology, Pathology and Laboratory Medicine, Aga Khan University Hospital, Karachi, PAK; 2 Histopathology, Aga Khan University Hospital, Karachi, PAK

**Keywords:** eccrine, spiradenoma, spiradenocarcinoma, sarcomatous, heterologous

## Abstract

Malignant eccrine spiradenoma (MES) is an exceedingly rare skin adnexal tumor that arises from pre-existing benign eccrine spiradenoma (BES). MES tumors show a wide spectrum of morphological features, posing a diagnostic challenge to the pathologist. Sarcomatous (heterologous) elements are seen in a few of these tumors, further complicating the morphological picture.

We herein describe a case of a 66-year-old male who presented with a recently enlarging, ulcerated, nodular skin lesion over the right leg that had been present for the last 25 years. The patient underwent wide local excision of the tumor.

Microscopic examination revealed a neoplastic lesion comprising benign and malignant components. The carcinomatous component showed features of infiltrating adenocarcinoma, not otherwise specified, whereas the sarcomatous component showed predominant osteosarcomatous and focal chondrosarcomatous differentiation. The benign component showed morphological and immunohistochemical features of BES. No adjuvant treatment was administered. The patient was alive and disease-free for 14 months, after which he was lost to follow-up.

Careful identification and knowledge related to histological diversity are keys to the correct diagnosis of this rare tumor. MESs are potentially aggressive tumors, and therefore, close long-term follow-up should be maintained.

## Introduction

Malignant eccrine spiradenoma (MES) is an extremely rare malignant adnexal neoplasm that arises from eccrine spiradenoma (ES) and accounts for 0.005% of all cutaneous neoplasms [1,2]. MES commonly occurs in elderly patients, and it exhibits a predilection for the head and neck region [1,3-6]. Patients usually present with the recent development of bleeding, ulceration, or rapid growth of a longstanding dermal nodular lesion [2-5,7-9]. MES can be sporadic (solitary lesion) or a component of Brooke-Spiegler syndrome (BSS) [1,3,5,6]. The malignant component of MES shows a wide spectrum of histological features, and it can be divided into low-grade and high-grade subgroups [3-6,8-10]. The presence of heterologous elements is extremely rare [1,5,10-12]. Because of its rarity and diversity of histological features, MES can pose diagnostic challenges, and it can be confused with other malignancies [3-6,8-10].

MESs exhibit potentially aggressive behavior. According to some authors, prognosis is related to histological grade because distant metastasis and tumor-related death are seen only in high-grade tumors [3-5]. However, patients with low-grade tumors experience local recurrence [3-6]. Patients with the non-metastatic disease have 100% disease-free survival with complete surgical excision; patients with lymph node and distant metastasis have poor survival, and they may benefit from a lymphadenectomy and adjuvant treatment, respectively [8]. We describe the clinicopathological and histological features of an MES case along with a review of the literature with the intent of describing features helpful in making the correct diagnosis and better understanding the disease behavior.

## Case presentation

A 67-year-old male presented to the general surgery clinic with a complaint of a nodular, ulcerative lesion on his right leg. The lesion had been present for 25 years, and it had suddenly increased in size, ulcerated, and become tender. The patient had no significant past medical or surgical history during the past 25 years. On examination, a raised nodular lesion with surface ulceration and bleeding was identified on the anterolateral aspect of the right leg. The lesion was tender, the overlying skin was partially mobile, and the tumor was freely mobile over the tibia. The inguinal and popliteal lymph nodes were not palpable. The clinical differential diagnosis was malignant neoplasm of cutaneous or skin adnexal origin. Computed tomography scans of the chest, abdomen, and pelvis did not reveal any metastatic disease. The patient underwent wide local excision of the tumor.

On gross examination, a vaguely circumscribed, gray-white, firm tumor was identified in the dermis and subcutaneous fat. The tumor measured 6 x 3 x 2.5 cm; it was limited on the deeper aspect and by the fascia.

**Figure 1:**
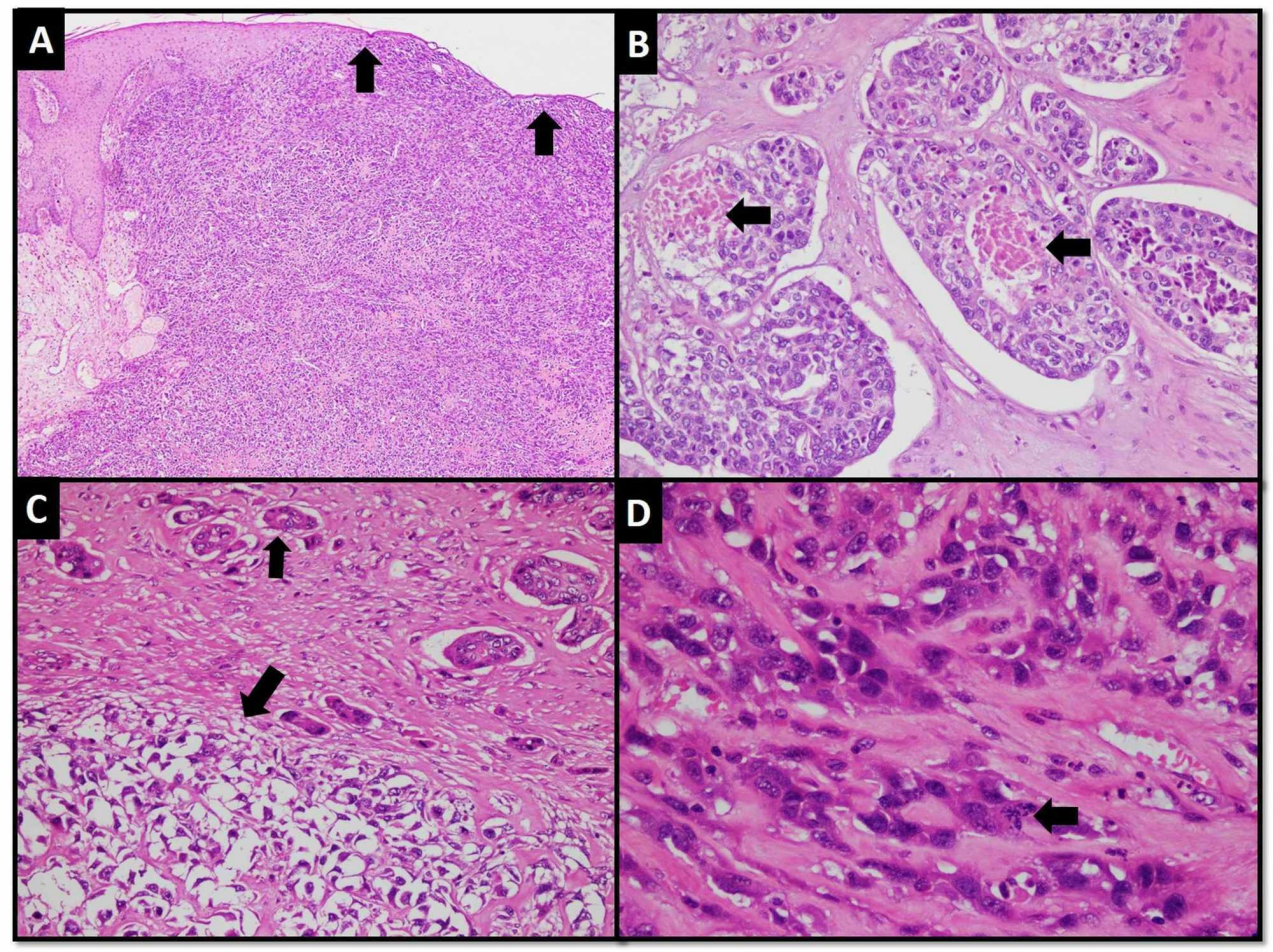
Carcionomatous component. (A) Low power view of tumor in the dermis showing sheet-like arrangement. Tumor is pushing the overlying skin and causing its thinning (arrows). (B) Tumor nests with central necrosis (arrows). (C) Tumor cells showing cytoplasmic clearing (larger arrow) and focal tubule formation (smaller arrow). (D) High power view of tumor cells showing moderate to markedly pleomorphic nuclei and mitotic figure (arrow).

Microscopic examination revealed a skin-covered tissue exhibiting a vaguely circumscribed neoplastic lesion comprising benign and malignant components that showed abrupt transitions. The carcinomatous component was arranged in the form of sheets, large nests, and clusters with focal tubule formation. Focal cytoplasmic clearing and focal squamoid change were also observed. The nuclei showed marked nuclear pleomorphism, vesicular, and frequent mitoses (approximately 14 mitoses per 10 high power fields {HFPs}). Foci of necrosis were also seen at the center of the tumor nests (Figure 1A to 1D).

**Figure 2:**
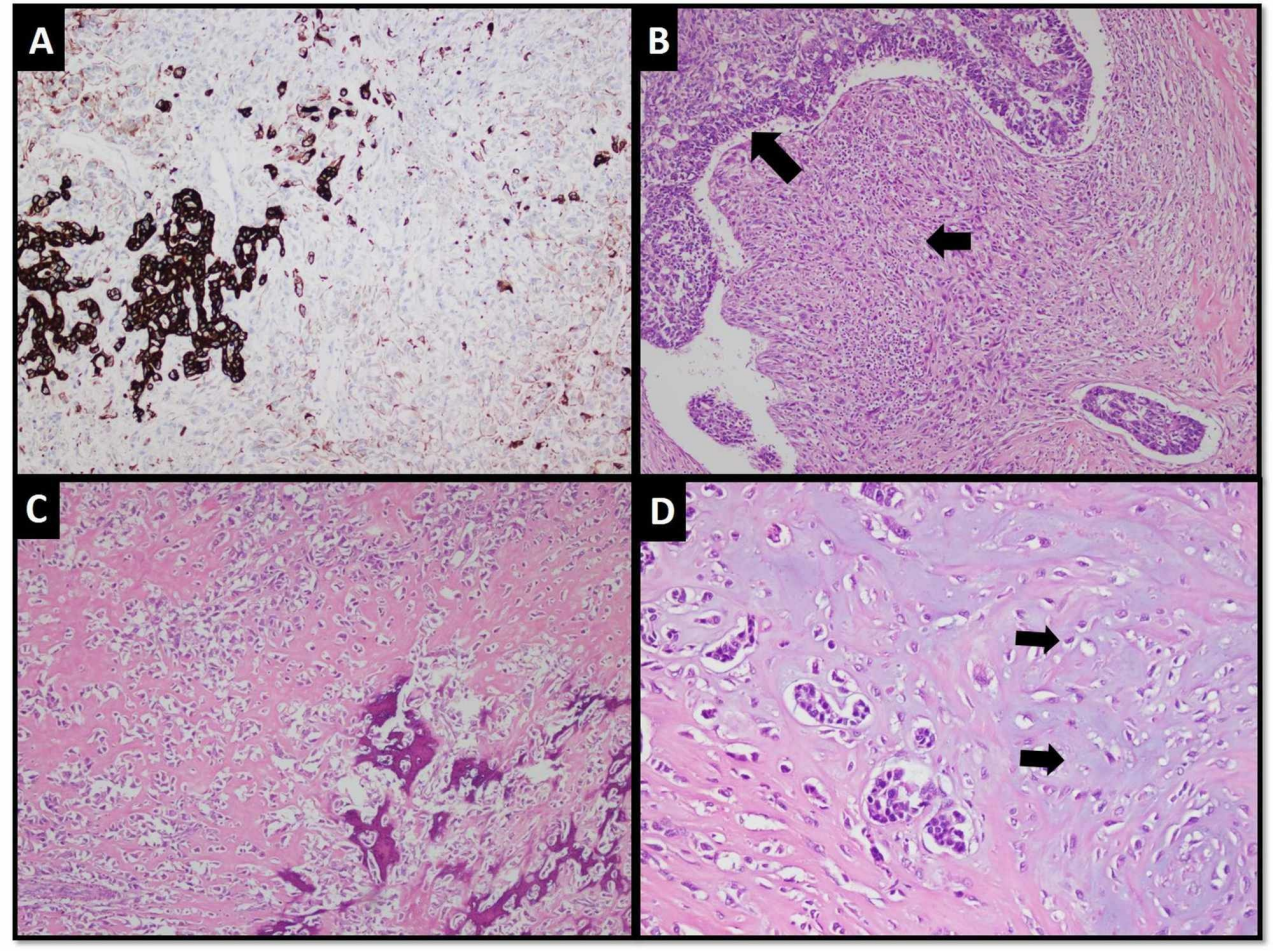
(A) Carcinomatous component demonstrating positive expression for cytokeratin AE1/AE3 IHC stain. (B) Nests of carcinomatous component (larger arrow) showing abrupt transition with pleomorphic sarcomatous component (smaller arrow). (C) Osteosarcomatous differentiation. (D) Chondrosarcomatous differentiation (arrows). IHC: immunohistochemical

These cells demonstrated positive expression for cytokeratin AE1/AE3 and cytokeratin 7 immunohistochemical (IHC) stains (Figure 2A). The carcinomatous component showed overall features of infiltrating adenocarcinoma, not otherwise specified; moreover, it showed both abrupt and gradual transitions with the sarcomatous component, which predominantly exhibited pleomorphic sarcoma-like appearance (Figure 2B). Focal osteosarcomatous differentiation characterized by deposition of extracellular osteoids by pleomorphic neoplastic cells was seen (Figure 2C). At foci, chondrosarcomatous differentiation was also observed (Figure 2D).

**Figure 3:**
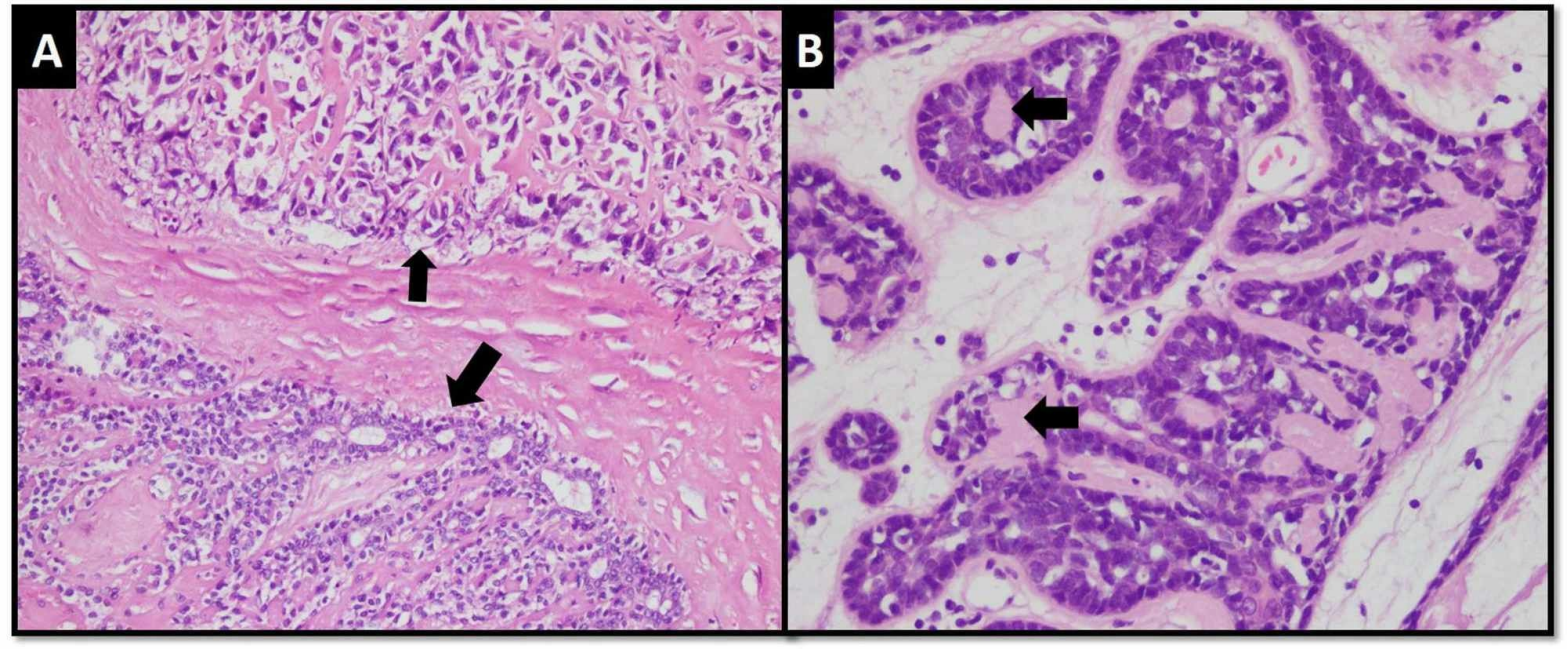
(A) Transition between benign component (larger arrow) and sarcomatous component (smaller arrow). (B) Benign component exhibiting trabeculae and tubules of basaloid cells along with larger cells having clear cytoplasm. Basal lamina-like material is also appreciable (arrows).

The benign component was present as discrete nodules at the tumor periphery, and it showed trabeculae and tubules of small basaloid cells intermixed with intermediate size clear cells. These cells showed mild pleomorphism, but no mitotic activity was appreciated. Deposition of basal lamina-like material and lymphocyte sprinkling were also observed (Figure 3A, 3B).

The benign component demonstrated positive expression for cytokeratin AE1/AE3, cytokeratin 7, alpha-smooth muscle actin (ASMA), S-100, and glial fibrillary acidic protein (GFAP) IHC stains. Hence, the diagnosis of MES was confirmed. All the margins were tumor-free.

The patient recovered well from the surgery. He did not receive any adjuvant chemotherapy or radiotherapy. He remained alive and disease-free for 14 months, after which he was lost to follow-up.

## Discussion

ES and cylindroma are closely related eccrine tumors that represent the ends of a spectrum, while spiradenocylindroma is a hybrid form of these two tumors [3,6]. Most of these tumors arise sporadically, and few of them are seen in the setting of BSS, which is an autosomal dominant syndrome comprising multiple dermal tumors, including cylindroma, ES, spiradenocylindroma, and trichoepithelioma [3,5,6]. MES, malignant cylindroma, and malignant spiradenocylindroma are extremely rare malignant tumors that arise from pre-existing benign tumors. Due to the extreme rarity and close relation between these malignant tumors, most of the literature regarding these tumor entities is either in the form of reports of a single case or small series comprising MES, malignant cylindroma, and malignant spiradenocylindroma [3,5,6,8]. A recent World Health Organization (WHO) classification of skin tumors has described these rare tumors as a single group and assigned them a unified International Classification of Disease for Oncology (ICD-O) code [1].

Malignant eccrine tumors occur in patients over a wide age range of 34-92 years [2-7,9,11,13,14]. The median age in different studies has been in the range of 60-72 years [1,8,15]. Tumor size has been reported to be in the range of 0.5-17.5 cm [3-7,10,12,14]. The age at presentation and tumor size of our patient was in concordance with the published literature. In most studies, no gender predilection has been observed [1,3,4,6]. The average duration of symptoms has been two decades, while the shortest and the longest durations reported in the literature are 7 months and 75 years [2-5,7,9]. Most cases reported in the study by Granter et al. had symptoms for two years or less [4]. The head and neck region represents the most common tumor site, followed by the trunk and extremities. Upper extremities are more commonly involved compared with lower extremities [1-7,9-11,14]. Clinically, these tumors usually appear during puberty and adolescence as skin nodules that may be associated with erythema and/or remain stable for decades. Patients with these tumors usually seek medical advice when they notice rapid increases in tumor size, pain, color change, ulceration, and bleeding [3-5,7,11]. Clinical differential diagnoses include melanoma, dermatofibrosarcoma protuberans, and epidermal inclusion cysts [7,8,14].

Microscopically, the presence of BES, cylindroma, or spiradenocylindroma is the prerequisite for the diagnosis of these malignant tumors [3]. The benign component can be found as a minute focus or comprise up to 90% of the tumor [4,6]. Granter et al. found that low-grade tumors exhibited gradual transitions, while high-grade tumors showed abrupt transitions [4]. The gradual transition of benign components into adenocarcinoma in situ and infiltrating adenocarcinoma with intervening atypical adenomatous areas explains the origin of these malignant tumors from their benign precursors [3,5].

In a study on low-grade tumors, well-demarcated and multinodular architecture was observed at low magnification [6]. However, other studies on low- and high-grade tumors identified lobular architectures and infiltrative patterns in almost equal numbers of cases [3,4].

The malignant component exhibits a wide variety of morphological features, ranging from low-grade carcinoma (resembling salivary gland-type low-grade basal cell adenocarcinoma {BCAC-LG}) to high-grade carcinoma resembling various cutaneous and non-cutaneous malignancies, such as salivary gland-type high-grade basal cell adenocarcinoma (BCAC-HG), infiltrating adenocarcinoma-not otherwise specified (IAC-NOS), malignant mixed tumor, squamous cell carcinoma, and renal cell carcinoma. These tumors should be considered in the differential diagnosis of MES. The morphological features of the carcinomatous component in our study resembled the features of IAC-NOS. Tumor grades and morphological patterns can be seen either exclusively or as a combination in individual tumors [3-6,8,9]. Low-grade (LG) tumors are characterized by rounded nodules, nests, and tubules of small to medium-sized basaloid cells with mild to moderate pleomorphism, vesicular nuclei with inconspicuous nucleoli, and low mitotic counts. Atypical mitoses, lymphovascular invasion (LVI), and perineural invasion have not been identified [1,3,8,16]. In one study, the mitotic count in LG tumors ranged from two to 14, with a mean of 6.3 mitoses/10 HPFs [4]. High-grade (HG) tumors are composed of infiltrative sheets, nests, and trabeculae of medium-sized to large cells exhibiting moderate to marked pleomorphism, nuclear hyperchromasia, and frequent mitoses along with atypical mitoses. Necrosis is frequently observed [1,3,4,5]. Mitotic counts in HG tumors can range from two to 62 mitoses/10 HPFs [3,4]. Tumors of both grades can show diverse histologic features such as squamous morules, keratocysts, clear cell change, apocrine secretions, mucinous metaplasia, oncocytic change, abundant extracellular myxoid material, extensive cystic change, psammomatous calcifications, stromal calcification, ectatic blood vessels, and dilated lymphatic spaces in a subset of cases [3-6,14]. IHC markers are of limited value in these tumors. Cytokeratins, epithelial membrane antigen (EMA), and carcinoembryonic antigen (CEA) IHC stains are frequently positive [1,6,7,9]. Gross cystic disease fluid protein-15 (GCDFP-15) IHC expression is observed in areas of apocrine differentiation [3].

The presence of sarcomatous components with heterologous differentiation in a subset of tumors exacerbates the diagnostic challenge. Heterologous differentiation observed in MES includes chondrosarcomatous, rhabdomyosarcomatous, osteosarcomatous, and leiomyosarcomatous differentiation [1,5,11,12]. The sarcomatous components in our case showed osteosarcomatous and chondrosarcomatous differentiation. Chou et al. summarized the clinicopathological features of 12 MES cases with sarcomatous components. Osteosarcomatous differentiation was observed in two cases, and osteocartilaginous, chondrosarcomatous, rhabdomyoblastic, and leiomyosarcomatous differentiation was observed in a single case each. In seven cases, sarcomatous components did not exhibit any heterologous differentiation [11]. In a study of 24 cases published by Kazakov et al., a sarcomatous component was observed in seven cases in the form of pleomorphic sarcoma. Two of these seven cases also showed areas of chondrosarcomatous differentiation, two showed spindle cell sarcoma, and one showed rhabdomyosarcomatous differentiation [5].

The diagnostic challenge in low-grade tumors is the identification of malignant components and their discrimination from benign components, while the challenge in high-grade tumors is the identification of benign components, which requires adequate sampling [4-6,8]. Histological features of malignant transformation include loss of dual cell population, loss of jigsaw or mosaic arrangement pattern, loss of peripheral hyaline sheath, sheet-like pattern, infiltrative borders, tumor necrosis, LVI, nuclear pleomorphism, hyperchromasia, and increased mitoses [3,5]. A few other features of malignancy encountered in a subset of cases include squamous morules, malignant mixed tumor-like areas, sarcomatoid carcinoma, and heterologous differentiation [3]. The malignant component exhibits loss of the myoepithelial cell layer, which is highlighted by loss of p63 IHC expression, partial or complete loss of the periodic acid Schiff positive hyaline sheath, and variable expression for p53 IHC stains [3,7,9]. In low-grade spiradenocarcinoma, p53 IHC expression of benign and malignant components does not differ significantly. However, the Ki-67 proliferative index of the malignant component is higher than that of the benign component. In one study, the Ki-67 indexes of benign components were in the range of 0-11%, and the Ki-67 indexes of malignant components were in the range of 0-24%. This difference was found to be statistically significant [6]. In another study, the Ki-67 proliferative indexes of the malignant component were in the range of 15-50%, and they were considerably higher than those in the benign component (values not mentioned) [3]. The malignant component showed significant loss of MYB IHC expression, while intact staining was observed in the benign component [6].

In a study of 19 cases of low-grade spiradenocarcinoma, local recurrence was observed in three cases, and none of the patients developed distant metastasis or tumor-related death [6]. In a study of 24 cases by Kazakov et al., among six patients with tumors exhibiting exclusive BCAC-LG morphology, local recurrence was observed in three cases, and none of the patients developed distant metastasis or died of the disease. Among six patients with tumors exhibiting exclusive BCAC-HG morphology, three developed distant metastasis and died of the disease. One of the patients with exclusive IAC-NOS morphology also developed distant metastasis and was living with the disease. One of the five patients with sarcomatous components died of the disease [5]. According to the literature review by Tay et al., local recurrence was observed in 57% of cases, and 39% of cases developed distant metastasis and died of disease [17]. According to another literature review by Requena et al., 12 of 33 patients with spiradenocarcinoma and 11 of 31 with cylindrocarcinoma developed distant metastasis. Fourteen of these 23 patients died of disease [18]. Granter et al. reported a series of 12 cases in which distant metastasis to lymph nodes was observed in only a single case with high-grade carcinoma after five years; tumor-related death was not observed in this study. The researchers attributed the high rate of distant metastasis in the other studies to the bias of reporting cases with aggressive behavior [4]. The findings of other studies suggest that tumors with high-grade malignant components behave aggressively and result in distant metastasis and tumor-related death, while tumors with low-grade malignant components exhibit local recurrence. Distant metastasis and tumor-related deaths are not observed in this subgroup of patients [3-6]. Tanese et al. reviewed the literature and found that the patients with tumors showing sarcomatous and squamous change had a significantly higher death rate [7]. However, in another study, only one out of five cases with sarcomatous components died of disease [5].

Due to the rarity of the tumors described here, there is no definitive consensus on the appropriate treatment [8]. Wide local excision of tumors with free margins is the mainstay of treatment. Regional lymphadenectomy may be performed in cases with suspected lymph node metastasis. Adjuvant chemotherapy and radiotherapy have been administered in a few cases, but a definitive role of adjuvant treatment has not yet been established [2-4,8,9]. These tumors usually metastasize to regional lymph nodes before they spread to the lung, liver, brain, spinal cord, and bone [2,8,9,13]. In a meta-analysis of 72 cases, lymph node metastasis and distant metastasis were associated with significantly reduced survival. In 35 patients without metastatic disease who underwent complete local surgical resection, the disease-free survival rate was 100% (mean follow-up duration: 33 months). Seven out of 12 patients with lymph node metastasis underwent additional lymphadenectomy, and six were disease-free (mean follow-up duration: 47 months). Three of the five patients who did not undergo lymphadenectomy died of metastatic disease (mean survival duration: 45 months). The median survival duration of 24 patients with distant metastasis was 16 months. Thirteen patients were treated with surgery alone, and the median survival duration was 12 months. Eleven patients were treated with adjuvant chemo and/or radiotherapy and the median survival duration was 20 months. The authors concluded that non-metastatic tumors had excellent outcomes and that complete surgical excision is sufficient treatment for these patients. In patients with lymph node metastasis, lymph node dissection provides a survival benefit. The survival duration was increased in patients with metastatic disease who received adjuvant treatment. Lack of statistical significance was attributed to the small sample size [8].

Patients with BSS demonstrate mutations of the CYLD gene located on chromosome 16q. In this study, one out of two cases of BSS (in which DNA material was amplified) demonstrated a novel germline mutation in the CYLD gene. None of the four sporadic cases tested for tumor and germline mutations revealed any CYLD gene mutation [5].

The basic limitation of this study is its short follow-up duration of only 14 months. Lack of molecular studies is another limitation of this study.

## Conclusions

MES can exhibit a spectrum of histological features, including sarcomatous heterologous elements, which increases the diagnostic difficulty of this rare neoplasm. Careful identification of both benign and malignant components and thorough knowledge about the diversity of histological features are necessary to reach an accurate diagnosis. Close long-term follow-up is recommended because recurrence and metastasis are common in these tumors. Adjuvant treatment might be considered in high-grade tumors.
